# LigaSure™ Hemorrhoidectomy for Complex Grade III Hemorrhoidal Disease: A Prospective, Single-Center, Cohort Study

**DOI:** 10.7759/cureus.110895

**Published:** 2026-06-15

**Authors:** Samer Al Hakkak

**Affiliations:** 1 Surgery, College of Medicine, University of Alkafeel, Najaf, IRQ

**Keywords:** circumferential prolapse, colorectal surgery, dearterialization, excisional hemorrhoid surgery, grade iii haemorrhoids, intermittent prolapse, ligasure device

## Abstract

Background: Grade III hemorrhoidal disease (HD) with large-volume or circumferential prolapse is poorly managed with less invasive treatments. This study evaluates LigaSure™ (Covidien plc, Dublin, Ireland; now acquired by Medtronic plc, Galway, Ireland) hemorrhoidectomy outcomes in this subset.

Methods: This was a prospective single-center cohort study conducted in Najaf Province, Iraq, which enrolled 316 adults with Grade III HD and at least one "special" feature (large solitary prolapse ≥3 cm or ≥1 quadrant, circumferential prolapse ≥3 quadrants, perianal fibrosis/edema, or prior failed rubber band ligation (RBL)/dearterialization (DEART)/stapled hemorrhoidopexy (SH)), from February 2023 to October 2024, with a follow-up to October 2025. Primary endpoints included operative time and 30-day complications (visual analog scale ≥7 pain, urinary retention, bleeding, infection, stenosis, incontinence). Secondary outcomes included length of stay (LOS), recovery time, and one-year recurrence.

Results: Median age of participants was 30 years (range 18-55). Of the total participants, 62.7% were male, and 48.1% had prior failed therapy. Mean operative time was 28.0 ± 6.0 minutes. Severe pain was recorded in 6.9% (95% CI 4.4-10.3), urinary retention in 17.1% (95% CI 13.1-21.7); the rate of postoperative urinary retention was higher with spinal (19.6%, n=240) versus general anesthesia (9.2%, n=76). The risk difference was 12.5% (95% CI 3.2-21.8). No bleeding, infection, stenosis, or incontinence was seen. Median LOS was one day (IQR 1, 1), and median recovery duration was seven days (IQR 6, 9). The one-year follow-up rate was achieved in 91.5% (289/316) of the patients. There were no patients with reoperation, and 11.8% had minor bleeding (95% CI 8.3-16.1). Outcomes were similar regardless of prior treatment failure.

Conclusion: Standardized LigaSure hemorrhoidectomy for complex Grade III HD demonstrated rapid operative times, low severe pain, acceptable urinary retention (higher with spinal anesthesia), and zero one-year reoperations, supporting early excisional surgery in this anatomical subset.

## Introduction

Hemorrhoidal disease (HD) is a prevalent condition, affecting approximately 4.4% of the population, with peak incidence between 45 and 65 years of age [1,2]. HD may also develop earlier, particularly in individuals exposed to dietary and lifestyle risk factors. Recognized risk factors include constipation, a low-fiber diet, an elevated body mass index (BMI), pregnancy, and a sedentary lifestyle [3]. Clinical management of HD is evidence-based, with both conservative and surgical treatment modalities established. Disease severity, as classified by the Goligher system, typically guides the selection of non-surgical or operative management, supported by various adjunctive therapeutic guidelines [4-6].

Grade III hemorrhoids are characterized by intermittent prolapse that is manually reducible and present with a broad spectrum of clinical phenotypes, each responsive to various therapeutic options. These include outpatient therapies such as rubber band ligation (RBL) and sclerotherapy, minimally invasive procedures like dearterialization (DEART) and stapled hemorrhoidopexy (SH), as well as conventional excisional hemorrhoidectomy. Less invasive treatments are associated with higher failure rates in cases of anatomically diverse conditions, such as large solitary piles or circumferential prolapses that may closely resemble Grade IV at defecation but remain reducible [4-6].

LigaSure™ (Covidien plc, Dublin, Ireland; now acquired by Medtronic plc, Galway, Ireland) is increasingly popular for hemorrhoidectomy, especially regarding operative time reduction and minimizing postoperative pain and bleeding compared with diathermy techniques [7-9]. However, data on the benefits of LigaSure in the Complex Grade III HD subtype remain limited. This single-center, prospective study was conducted to evaluate the safety and efficacy of LigaSure hemorrhoidectomy (LSH) in patients with large-volume Complex Grade III HD, with particular attention to comparability with existing LSH literature in Grade III disease and outcomes in patients with prior failed conservative or minimally invasive therapy.

## Materials and methods

Study design and setting

This was a prospective, single-center observational clinical study conducted at Najaf Hospital and its affiliated surgical outpatient clinics in Najaf Province, Iraq. Patient enrollment was completed between February 1, 2023, and October 31, 2024, and collection of all one-year follow-up data was finalized in October 2025. The study was approved by the Ethics Committee of Alkafeel University, Republic of Iraq (approval number: 3, dated January 21, 2023), and fully complied with the STROBE (Strengthening the Reporting of Observational Studies in Epidemiology) guidelines [10].

Study population

Inclusion and Exclusion Criteria

Patients were eligible for inclusion if they presented with symptomatic Complex Grade III HD, defined as intermittent prolapse requiring manual reduction alongside specific large-volume features. These qualifying features included: (i) A prominent single prolapse measuring at least 3 cm in maximum dimension or encompassing a complete quadrant (90 degrees) at top stress, (ii) A circumferential prolapse involving three or more quadrants (≥270 degrees) at maximal strain, (iii) The presence of perianal fibrosis or edema, clinically assessed as thickening, induration, or edematous perianal skin, or (iv) Continuing symptoms due to the failure of prior conservative or minimally invasive treatments, such as RBL (defined as persistence or recurrence of symptoms after ≥2 sessions), DEART (defined as persistence or recurrence after one procedure), or SH (defined as persistence or recurrence after one procedure). The interval between the last failed procedure and LSH enrollment was required to be ≥3 months to allow for adequate healing and assessment of treatment failure.

Patients were excluded from the study if they presented with Grade IV HD (non-reducible prolapse), acute thrombosed hemorrhoids, inflammatory bowel disease, or immunosuppression. Further exclusion criteria included coumadinism or continuous anticoagulation therapy, pregnancy, and a history of a previous excisional hemorrhoidectomy.

Sample Size

All patients who presented to the surgical outpatient clinics with symptomatic HD during the study period were evaluated. Consecutive eligible patients who provided written informed consent were included using a convenience sampling approach over the 20-month enrollment period. No formal a priori sample size calculation was conducted due to feasibility constraints and the absence of prior data specifically for the Complex Grade III HD subtype; an interpretability sample approach was used, resulting in a final cohort of 316 included patients.

Operative technique (LigaSure hemorrhoidectomy)

All surgical procedures were performed at Najaf Hospital by one of three consultant colorectal surgeons (each with >10 years of subspecialty experience). Patients were positioned in the lithotomy position and administered either spinal anesthesia (0.5% bupivacaine, 12-15 mg) or general anesthesia, depending on patient preference and surgeon discretion. A Lone Star® Retractor System (CooperSurgical Inc., Trumbull, Connecticut, United States) was utilized to expose the hemorrhoidal cushions. The procedure was carried out using the LigaSure LF2019 Exact Dissector vessel-sealing device (seal length 20.6 mm), configured to the "hemorrhoid" setting. The operative technique involved pushing, lifting, and subsequently sealing and cross-clamping the pedicle at the base of the hemorrhoid without any prior infiltration of adrenaline. Standard postoperative packing was not utilized in any of the cases.

Anesthetic and analgesic protocols

Anesthesia allocation was non-randomized and determined by patient preference and surgeon discretion based on clinical assessment of patient factors (e.g., comorbidities, anxiety, anticipated procedure duration). Postoperative analgesia followed a standardized protocol: oral acetaminophen 1 g every six hours for 48 hours, with rescue tramadol 50 mg every eight hours as needed. Parenteral opioids (morphine 5-10 mg intramuscularly) were reserved for patients with visual analog scale (VAS) pain scores ≥7 despite oral analgesia.

Follow-up protocol

Follow-up visits were scheduled at one week, one month, three months, six months, and 12 months postoperatively. At each visit, a trained research nurse (blinded to prior treatment status) assessed symptoms, examined the surgical site, and recorded complications using standardized case report forms. Between scheduled visits, patients were contacted by telephone at two weeks and nine months to assess interim symptoms and satisfaction. The one-year follow-up visit included digital rectal examination and anoscopy by the operating surgeon to assess for recurrence, stenosis, and continence. Patient satisfaction was assessed using a single-item question: "How satisfied are you with the outcome of your surgery?" with response options: very satisfied, satisfied, neutral, dissatisfied, very dissatisfied.

Definition

To standardize terminology, "Complex Grade III HD" is defined in this study as large-volume Grade III disease characterized by prominent single prolapse, circumferential prolapse, significant perianal fibrosis or edema, or cases refractory to previous conservative or minimally invasive treatments. Inter-observer agreement for the classification of these complex features was assessed prospectively using the kappa statistic (κ = 0.84, 95% CI 0.76-0.92), indicating substantial agreement between the three participating consultant colorectal surgeons.

Statistical analysis

Data analysis was performed using IBM SPSS Statistics for Windows, version 26 (Released 2018; IBM Corp., Armonk, New York, United States). Continuous variables are presented as the mean ± standard deviation (SD) or median (range/interquartile range (IQR)) as appropriate, while categorical variables are expressed as counts and percentages. Confidence intervals (CIs) for proportions were calculated using the exact binomial approach. Subgroup analyses were conducted to compare outcomes between patients with and without previous treatment failures. Categorical variables were compared using the Chi-square test or Fisher's exact test, while continuous variables were analyzed using the independent samples Student's t-test or the Mann-Whitney U test, depending on the normality of the data distribution. Statistical significance was defined as p < 0.05.

Given the absence of an a priori power calculation, we performed post hoc power analyses for key binary outcomes. For the primary safety outcome of severe pain (observed rate 6.9%), the study had 80% power to detect a 5% absolute difference from a hypothesized 12% rate. For rare complications (stenosis, incontinence), the observed zero events provide reassurance but must be interpreted with caution given limited statistical power to exclude low-incidence events.

## Results

This extensive, prospective single-center study enrolled 316 patients, constituting one of the largest cohorts to date evaluating LigaSure hemorrhoidectomy specifically for Complex Grade III HD. Baseline demographic and clinical characteristics are summarized in Table 1. The median age was 30 years (range 18-55), with 62.7% male participants. Notably, 81.6% reported constipation (<3 bowel movements per week), 91.5% reported prolonged toilet time (>10 minutes), and the mean BMI was 27.4 ± 4.2 kg/m².

**Table 1:** Baseline Demographic and Clinical Characteristics (N=316)

Characteristic		Value
Age (years), median (range)		30 (18-55)
Sex, n (%)	Male	198 (62.7%)
	Female	118 (37.3%)
Body Mass Index (kg/m²), mean ± SD		27.4 ± 4.2
Constipation (<3 bowel movements/week), n (%)		258 (81.6%)
Diarrhea (frequent episodes), n (%)		48 (15.2%)
Prolonged toilet time (>10 min), n (%)		289 (91.5%)

Anatomical and clinical characteristics defining the "complex" phenotype are presented in Table 2. Circumferential prolapse (≥3 quadrants) was the most common feature (79.7%), followed by large single prolapse (69.0%), perianal fibrosis or edema (49.4%), and prior failed treatment of any type (48.1%). Among patients with prior failed therapy, RBL was the most common (n=89, 28.2%), followed by DEART (n=42, 13.3%) and SH (n=21, 6.6%).

**Table 2:** Anatomical and Clinical Characteristics Defining Complex Grade III HD

Feature	Frequency (Percentage)
Large single prolapse (≥3 cm or ≥1 quadrant)	218 (69.0%)
Circumferential prolapse (≥3 quadrants)	252 (79.7%)
Perianal fibrosis/edema	156 (49.4%)
Prior failed treatment (any)	152 (48.1%)
Rubber band ligation	89 (28.2%)
Dearterialization	42 (13.3%)
Stapled hemorrhoidopexy	21 (6.6%)

Primary outcomes, including perioperative parameters and immediate postoperative complications, are detailed in Table 3. The mean operative time (skin-to-skin) was 28.0 ± 6.0 minutes. Severe postoperative pain, defined as a visual analog scale (VAS) score ≥7 necessitating parenteral opioid administration, was observed in 6.9% of patients (95% CI 4.4-10.3). Urinary retention requiring catheterization was documented in 17.1% of the cohort (95% CI 13.1-21.7). Stratified analysis demonstrated a significantly higher incidence of urinary retention among patients receiving spinal anesthesia (χ² = 8.72, p = 0.003), as further specified in the table by anesthesia modality. No cases of postoperative bleeding requiring intervention, infection requiring extended antibiotics, stenosis, or new-onset fecal incontinence were reported.

**Table 3:** Perioperative Parameters and Early Postoperative Complications (30-Day) Data given as frequency (percentage) except for Operative time, which is given as mean ± SD, and Time to normal activities and Length of stay, which are given as median (IQR) IQR: interquartile range

Outcome	Frequency (Percentage)	95% CI
Operative time (minutes), mean ± SD	28.0 ± 6.0	—
Length of stay (days), median (IQR)	1 (1-1)	—
Severe pain (VAS ≥7, requiring parenteral opioids)	22 (6.9%)	4.4–10.3
Urinary retention (requiring catheterization)	54 (17.1%)	13.1–21.7
Spinal anesthesia subgroup (n=240)	47 (19.6%)	14.8–25.2
General anesthesia subgroup (n=76)	7 (9.2%)	3.8–17.9
Bleeding requiring intervention	0 (0%)	0–1.2
Infection requiring >3 days of antibiotics	0 (0%)	0–1.2
Stenosis requiring intervention	0 (0%)	0–1.2
New-onset fecal incontinence	0 (0%)	0–1.2
Time to normal activities (days), median (IQR)	7 (6-9)	—

Late postoperative complications and one-year follow-up outcomes are presented in Table 4. Importantly, no cases of postoperative bleeding requiring intervention occurred, and no patients developed anal stenosis or fecal incontinence. At one year, the recurrence rate defined as prolapse requiring manual reduction or bleeding necessitating reintervention was 0%, with no reoperations performed. Minor, self-limited bright red bleeding occurred in 11.8% of patients (95% CI 8.3-16.1), and persistent constipation requiring laxatives was reported in 11.8% (95% CI 8.3-16.1). Patient satisfaction at one year was high, with 93.8% (95% CI 90.2-96.3) reporting a satisfied or very satisfied status.

**Table 4:** Mid-Term Clinical Outcomes at One-Year Follow-Up

Outcome	Frequency (Percentage)	95% CI
No recurrent prolapse requiring manual reduction	289 (100%)	98.7–100
Occasional minor bleeding (bright red, self-limited)	34 (11.8%)	8.3–16.1
Persistent constipation (requiring laxatives)	34 (11.8%)	8.3–16.1
Reoperation for recurrent prolapse	0 (0%)	0–1.3
Patient satisfaction at 1 year (satisfied/very satisfied)	271 (93.8%)	90.2–96.3

Subgroup analyses shown in Table 5 indicated that mid-term clinical outcomes did not differ significantly between patients with or without a history of failed conservative management: mean operative time (29.1 ± 6.2 vs. 27.3 ± 5.8 minutes, t = 1.76, p = 0.08), severe pain (7.2% vs. 6.7%, χ² = 0.036, p = 0.85), urinary retention (17.8% vs. 16.5%, χ² = 0.093, p = 0.76), and oned-year minor bleeding (12.5% vs. 10.9%, χ² = 0.204, p = 0.65) were all comparable between groups. These findings suggest that prior failure of conservative or minimally invasive therapy does not compromise subsequent LSH outcomes.

**Table 5:** Subgroup Comparison of Outcomes by Prior Treatment History Values given as frequency (percentage) except for Operative time, which is given as mean ± SD

Outcome	Prior Failure (n = 152)	No Prior Treatment (n = 164)	Test Statistic	p-value
Operative time (minutes), mean ± SD	29.1 ± 6.2	27.3 ± 5.8	t = 1.76	0.08
Severe pain (POD 1+)	11 (7.2%)	11 (6.7%)	χ² = 0.036	0.85
Urinary retention	27 (17.8%)	27 (16.5%)	χ² = 0.093	0.76
1-year minor bleeding*	18 (12.5%)	16 (10.9%)	χ² = 0.204	0.65
1-year recurrence (prolapse or bleeding requiring intervention)	0 (0%)	0 (0%)	—	—

## Discussion

Age distribution and demographics

The median age of 30 years in this cohort is notably younger than the reported peak incidence of HD (45-65 years) in Western populations [1,2]. This demographic difference may reflect regional variations, differences in dietary habits (such as lower fiber and hydration intake), genetic predispositions, or lifestyle characteristics prevalent in Middle Eastern populations. Additionally, younger patients with extensive, circumferential prolapse may have a lower tolerance for functional impairment, leading to earlier presentation for surgical evaluation. However, age alone should not dictate surgical timing; the decision to proceed with hemorrhoidectomy must be individualized, considering symptom severity, anatomical features, comorbidities, and patient preferences.

LSH outcomes

The findings of this study align with meta-analytic data demonstrating operative time savings and reduced postoperative pain and hemorrhagic complications associated with LigaSure hemorrhoidectomy compared to traditional diathermy techniques [7-9]. The mean operative time of 28 minutes was shorter than the average reported for Milligan-Morgan procedures (35-45 minutes) [11]. The severe pain rate of 6.9% also compares favorably with historical Milligan-Morgan cohorts [11]. Urinary retention occurred in 17.1% of patients, a complication commonly associated with spinal anesthesia during colorectal surgeries [12], which our stratified analysis confirmed (19.6% with spinal vs. 9.2% with general anesthesia). Importantly, this association must be interpreted with caution given the non-randomized anesthesia allocation; patients receiving spinal anesthesia may have differed systematically from those receiving general anesthesia in unmeasured factors such as comorbidity burden, anxiety, or anticipated procedure complexity.

Remarkably, no procedures were complicated by postoperative bleeding requiring intervention, and no reoperations for recurrence were necessary at one year. These outcomes are favorable when compared to reported recurrence rates for RBL (16% to 29%) [13], Doppler-guided hemorrhoidal artery ligation (3%-24%) [14], and SH (up to 30%) [15]. However, these comparisons are indirect and across different study populations, time periods, and follow-up durations; they should not be interpreted as evidence for superiority of LSH over these alternatives in all clinical scenarios.

Outcomes in patients with prior failed therapy

A key finding of this study is the comparable outcomes between patients with prior failed conservative or minimally invasive therapy and treatment-naïve patients. The absence of significant differences in operative time, pain, urinary retention, or one-year bleeding suggests that prior failure of RBL, DEART, or SH does not compromise subsequent LSH efficacy or safety. This is clinically relevant, as 48.1% of our cohort presented after unsuccessful prior treatment. However, these findings should not be interpreted as justification to bypass conservative or minimally invasive therapies as first-line treatment. The study design specifically enrolled patients who had either failed prior therapy or had anatomical features (large-volume or circumferential prolapse) making conservative management unsuitable. For treatment-naïve patients with less complex Grade III HD, established conservative and minimally invasive options remain appropriate initial strategies.

Patient selection and individualized decision-making

Proper timing of surgical intervention in each case is essential, depending on co-morbidities. The decision to proceed with LSH should be individualized based on: (i) Anatomical features: Large-volume prolapse (≥3 cm or ≥1 quadrant), circumferential involvement (≥3 quadrants), or significant perianal fibrosis/edema may render conservative therapies less effective or anatomically unsuitable, (ii) Symptom severity and quality of life: Patients with recurrent bleeding, significant pain, or hygiene difficulties despite conservative measures may benefit from surgical intervention, (iii) Prior treatment history: Patients who have failed ≥2 sessions of RBL, or one session of DEART or SH, with an interval of ≥3 months allowing for adequate healing and assessment, may be considered for LSH, (iv) Comorbidities and contraindications: Active anticoagulation, inflammatory bowel disease, immunosuppression, or pregnancy may preclude or delay surgical intervention (these were exclusion criteria in our study, and their presence in clinical practice requires individualized risk-benefit assessment), and (v) Patient preferences and expectations: Shared decision-making should incorporate patient values regarding recovery time, pain tolerance, and acceptance of procedural risks.

A tailored, individualized approach is preferable to uniform recommendations for early surgical intervention in all cases. The data from this cohort demonstrate that LSH is effective when appropriately indicated; they do not demonstrate that surgery should be indicated earlier than current evidence-based practice would support.

Strengths and limitations

The strengths of this study include its large, prospective single-center design, the use of objective criteria to define Complex Grade III HD with substantial inter-observer agreement (κ = 0.84), and a high one-year follow-up rate of 91.5%. The absence of anal stenosis and fecal incontinence is further testimony to strict surgical standardization by trained subspecialists.

However, this study has significant limitations that must be acknowledged. The absence of a randomized comparator arm is a major limitation. Without randomization to alternative treatments (e.g., conventional diathermy hemorrhoidectomy, continued conservative management) or to different surgical timing, causal inferences about the superiority of LSH or the optimal timing of intervention cannot be made. The study design does not allow conclusions regarding the optimal timing of surgical intervention. While outcomes were favorable in this cohort, we cannot determine whether earlier surgery would have produced better results than delayed surgery or continued conservative management. The decision to proceed with surgery and its timing should remain individualized based on patient anatomy, symptom severity, comorbidities, and preferences.

Anesthesia allocation was non-randomized and determined by patient preference and surgeon discretion. The observed association between spinal anesthesia and urinary retention (19.6% vs. 9.2%) may be confounded by unmeasured patient factors (e.g., comorbidity burden, procedural complexity, anxiety). Causation cannot be inferred from this observational comparison. The single-country setting (Iraq) and notably young median age (30 years) limit generalizability to populations with different demographic profiles, dietary habits, genetic factors, and healthcare access. The peak incidence of HD in Western populations is 45-65 years [1,2], and our cohort may not reflect the typical Western surgical population.

The one-year follow-up duration is insufficient to assess long-term recurrence, which typically requires three to five years of observation for accurate estimation. The 0% reoperation rate at one year is encouraging but may not reflect long-term recurrence. The 11.8% minor bleeding rate may indicate ongoing disease activity that could progress to symptomatic recurrence with longer follow-up. Convenience sampling may introduce selection bias, and the absence of an a priori power calculation limits the interpretation of rare complications. Zero events for stenosis and incontinence provide reassurance but must be interpreted with caution; the study may have been underpowered to detect low-incidence adverse events. Detection bias may exist in outpatient follow-up settings. Minor bleeding (11.8%) and patient satisfaction (93.8%) may be subject to under-reporting or social desirability bias.

The study lacks validated patient-reported outcome measures (PROMs) such as quality-of-life instruments. Patient satisfaction was assessed with a single-item question rather than validated disease-specific questionnaires. Furthermore, while mid-term outcomes are excellent, the follow-up period needs to be extended to three to five years for proper late recurrence assessment. Comparative randomized trials with longer follow-up are needed to establish the optimal position of LSH in the treatment algorithm for this distinct clinical entity.

## Conclusions

This single-center prospective cohort demonstrates favorable safety and efficacy outcomes for LSH in Complex Grade III HD. In this selected cohort, LSH for Complex Grade III HD appears safe and effective, including in patients with large-volume or circumferential intermittent prolapse. The procedure was associated with reduced operative times, low rates of severe pain, acceptable management of urinary retention (notably higher with spinal anesthesia), and no reoperations within the first year. Outcomes in patients with prior unsuccessful conservative management were comparable to those in treatment-naïve patients, suggesting that prior failure of conservative therapy does not compromise LSH efficacy. This supports LSH as a viable surgical option after conservative or minimally invasive management has failed, or when anatomical features make such treatments unsuitable. A tailored approach considering individual patient anatomy, symptom severity, comorbidities, and prior treatment history remains essential in clinical practice. The decision to proceed with surgery and its timing should be individualized, incorporating shared decision-making between surgeon and patient.
